# Association of Long-Term Proton Pump Inhibitor Use With Nutrient Deficiencies: A Retrospective Cross-Sectional Study

**DOI:** 10.7759/cureus.106137

**Published:** 2026-03-30

**Authors:** Sarah Nafea, Chinenye Iguh, Fariyah Waseem, Tooba Babar, Mohd Hamza Masood, Muhammad Ammar Ali, Fatima Bashir Ahmed, Haya Qasem, Noel George Cherian, Ghosoun Ibrahem Atoom, Ban Ibrahim Atoum

**Affiliations:** 1 Medicine, Basildon & Thurrock University Hospital NHS Foundation Trust, Essex, GBR; 2 Medicine, Windsor University School of Medicine, Cayon, KNA; 3 Medicine, Lancashire Teaching Hospitals NHS Foundation Trust, Preston, GBR; 4 Internal Medicine, Combined Military Hospital, Rawalakot, PAK; 5 Internal and Acute Medicine, Queen Elizabeth The Queen Mother Hospital, Margate, GBR; 6 Internal Medicine, International Higher School of Medicine, Bishkek, KGZ; 7 Medicine, Northern Lincolnshire and Goole NHS Foundation Trust, Scunthorpe, GBR; 8 Medicine, Jinnah Sindh Medical University, Karachi, PAK; 9 Medicine, Prime Hospital, Dubai, ARE; 10 Medicine, Gulf Medical University, Ajman, ARE; 11 General Practice, Thumbay University Hospital, Ajman, ARE; 12 Medicine, Yarmouk University, Irbid, JOR; 13 Medicine, Jordan University of Science & Technology, Irbid, JOR

**Keywords:** calcium deficiency, hypomagnesemia, iron deficiency, long-term therapy, nutrient deficiences, proton pump inhibitors, vitamin b12 deficiency

## Abstract

Background

Proton pump inhibitors (PPIs) are generally employed to treat acid-related disorders. However, chronic acid blockade can reduce the absorption of essential micronutrients, such as vitamin B12, magnesium, calcium, and iron, potentially contributing to clinically significant deficiencies as defined by standard laboratory reference ranges.

Methods

A retrospective observational study was conducted using electronic medical records collected between July and December 2025. The study included adults (18 years and older) who had been taking PPIs continuously for six months or more. Nutrient deficiencies were defined according to institutional laboratory reference ranges, with the following cut-off values: vitamin B12 <200 pg/mL, magnesium <1.7 mg/dL, calcium <8.6 mg/dL, and ferritin <30 ng/mL. Both centers used identical reference ranges and standardized laboratory protocols, and all results were cross-checked to minimize inter-laboratory variability; any inconsistent or repeated values were excluded from analysis. The associations between PPI type/duration and deficiency status were analyzed using the Fisher-Freeman-Halton exact test, the chi-square test, and the Kruskal-Wallis test (p<0.05).

Findings

The sample consisted of 255 participants: 139 (54.5%) female patients and 116 (45.5%) male patients. The patients were on omeprazole (n=101; 39.6%), pantoprazole (n=64; 25.1%), esomeprazole (n=61; 23.9%), and lansoprazole (n=29; 11.4%). The prevalence of vitamin B12 deficiency varied by PPI type: 39 (38.6%) of omeprazole users and 36 (59.0%) of esomeprazole users were vitamin B12 deficient (p=0.018). Users of esomeprazole had the highest prevalence of magnesium deficiency (n=24; 39.3%) vs users of omeprazole (n=12; 11.9%; p=0.004). Calcium deficiency according to the type of PPI also differed, wherein eight (7.9%) patients on omeprazole and nine (31.0%) patients on lansoprazole were affected (p=0.021); the median serum calcium was found to be between 9.06 mg/dL and 8.71 mg/dL (H=11.29, p=0.010). There were no significant differences in iron/ferritin deficiency according to the type of PPI (33/101 (32.7%) in omeprazole users vs 29/61 (47.5%) in esomeprazole users) or PPI duration (p≈0.12).

Conclusion

Long-term use of PPIs was associated with a high prevalence of micronutrient deficiencies, especially B12, magnesium, and calcium, with substantial differences by PPI type. These results highlight the importance of routine nutritional monitoring in patients receiving long-term PPI therapy.

## Introduction

Proton pump inhibitors (PPIs) are widely prescribed worldwide and are mainly used to treat acid-related gastrointestinal disorders, e.g., gastroesophageal reflux disease (GERD), peptic ulcer disease, and Zollinger-Ellison syndrome [1,2]. PPIs reduce the release of gastric acid by irreversibly binding to the H+/K+ ATPase enzyme of gastric parietal cells, thereby producing relief of symptoms and mucosal healing [3]. Although short-term PPI use is generally considered safe, long-term therapy has raised concerns due to potential adverse effects, including micronutrient deficiencies such as vitamin B12 deficiency and other risks such as cognitive decline, chronic kidney disease, bone fractures, and cardiovascular events [4,5].

Gastric acid is also necessary for the absorption of several essential nutrients, including vitamin B12, magnesium, calcium, and iron [6]. Prolonged inhibition of gastric acidity can also impair nutrient absorption, which, in turn, can result in nutrient deficiencies with clinical implications, including anemia, bone demineralization, and neurological problems [7]. Also, a large number of observational studies have exhibited that PPI users who have two or more years of drug exposure are more likely to be diagnosed with vitamin B12 deficiency compared to non-users, which supports this concept of long-term nutrient malabsorption [8,5]. Although there is increasing evidence on the clinical relevance of nutrient deficiencies associated with PPI use, such deficiencies have not yet been systematically examined in routine clinical practice in Pakistan, and the literature is generally limited by small sample sizes or by the lack of studies assessing long-term PPI use (defined as ≥six months of continuous therapy) [7,9].

This retrospective cross-sectional study aims to examine the association between long-term PPI use and nutrient deficiency in adult patients. The purpose of this study was to assess the prevalence of micronutrient deficiencies, including vitamin B12, magnesium, calcium, and iron, among adults receiving long-term PPI therapy.

Study rationale and novelty

PPIs are widely used in the treatment of acid-related disorders of the gastrointestinal system, and they are often used for longer than recommended. Although the treatment is effective in controlling symptoms, taking PPIs over time can cause problems because they can disrupt the absorption of essential nutrients, which can affect our overall health. Despite increasing recognition of the risks, data on the prevalence of nutrient deficiencies among patients on long-term PPI therapy in Pakistan are limited. Prior studies are constrained by small sample sizes and lack of stratification by PPI type and duration, leaving the broader prevalence and clinical relevance of these deficiencies unclear [7,9].

This study aims to fill this gap by investigating the association between long-term PPI use and various nutrient deficiencies among adults. The novelty of the research lies in its comprehensive foundation on essential nutrients in a patient population in a real-life setting, which may aid clinical observation, early identification of deficiencies, and informed decision-making regarding the type of PPI therapy. The results provide insight into the prevalence of micronutrient deficiencies among long-term PPI users, which may inform awareness and monitoring practices in clinical settings.

Study objectives

The primary objective of this study was to assess the association between long-term PPI use and micronutrient deficiencies among adult patients. Specifically, the study evaluated the prevalence of deficiencies in vitamin B12, magnesium, calcium, and iron among individuals receiving prolonged PPI therapy.

The secondary objective was to examine patterns of these deficiencies by PPI duration (≥six months) and patient characteristics, including age and sex, to identify potential high-risk subgroups.

## Materials and methods

Study design and setting

This retrospective observational study examined the association between long-term use of PPIs and the prevalence of nutrient deficiencies in adult patients. Data were retrospectively obtained from tertiary care centers and were pooled for analysis. Electronic medical records and laboratory databases were used to extract data between July 2025 and December 2025. The records included patients with a history of continuous PPI use for at least six months prior to data extraction. All laboratory tests and clinical information were collected in accordance with hospital guidelines. 

Sample size

The required sample size was estimated using the standard formula for prevalence studies:

\[n = \frac{Z^2 \cdot p (1 - p)}{d^2}\]

assuming a 95% confidence level, 5% margin of error, and an expected prevalence of 50% to yield the maximum sample size [10]. This calculation suggested 385 participants. However, due to feasibility and logistical constraints, the final study included 255 participants. Long-term PPI use was defined as continuous therapy for six months or more.

Inclusion and exclusion criteria

Adults aged 18 years and older with documented continuous PPI use for at least six months were included in the study. Continuous use was verified through electronic medical and pharmacy records, and adherence was assumed based on prescription refill history. Both male and female subjects were eligible. Patients with conditions that could directly interfere with nutrient absorption were excluded, including individuals with severe liver disease or documented malabsorption syndromes, identified through medical records, laboratory results, and physician documentation. Patients taking high-dose vitamin or mineral supplements - defined as more than 1.5 times the recommended daily allowance of vitamin B12, magnesium, calcium, or iron - were also excluded. Common comorbidities such as chronic kidney disease, hypertension, and diabetes were retained in the study population to reflect real-world clinical practice, although their potential confounding effects are acknowledged in the limitations section. Patients with any gaps in their laboratory records for the nutrients of interest were also excluded.

Demographics and clinical data

Medical records were reviewed, and patient demographic and clinical characteristics were abstracted using a structured data extraction form. The variables were age, sex, body mass index (BMI), smoking status, comorbid conditions, and concomitant medication. PPI-specific data were collected, including the type (omeprazole, pantoprazole, esomeprazole, lansoprazole); no other PPIs were reported in the study population. Duration of use (six to 12 months, one to three years, or more than three years), and dose (standard or high) were also assessed. Smoking status was categorized as current smoker, former smoker, or non-smoker based on documentation in the medical records. BMI was calculated as weight in kilograms divided by height in meters squared.

Laboratory assessment

Each patient had laboratory-confirmed nutrient deficiencies, as determined by vitamin B12, magnesium, calcium, and iron/ferritin levels. Nutrient deficiencies were defined according to institutional laboratory reference ranges: vitamin B12 <200 pg/mL, magnesium <1.7 mg/dL, calcium <8.6 mg/dL, and ferritin <30 ng/mL. Only laboratory results obtained during active PPI therapy and within six months prior to data extraction were included, to ensure relevance to long-term treatment. The six-month cut-off was chosen as a conservative window to capture nutrient status likely influenced by chronic PPI use while maintaining sufficient sample availability.

Outcomes

Primary Outcome

The primary outcomes of the study were the prevalence of deficiencies in vitamin B12, magnesium, calcium, and iron/ferritin among adults on long-term PPI therapy. 

Secondary Outcomes

Secondary outcomes included the association between PPI use duration and the development of nutrient deficiencies, as well as possible variation by sex, age, and comorbidity. The patterns of various nutrient deficiencies in the same patient were also examined in the study.

Statistical analysis

IBM SPSS Statistics for Windows, Version 26 (Released 2019; IBM Corp., Armonk, New York, United States) was used to analyze data. Descriptive statistics, such as frequencies and percentages, were used to summarize baseline characteristics of the study population, such as age, sex, BMI, comorbidities, and concomitant medications. Potential confounding by concomitant medications (e.g., metformin, diuretics) was not adjusted for in this analysis and is acknowledged as a limitation. The Shapiro-Wilk test was used to assess the normality of continuous variables, i.e., serum Vitamin B12, magnesium, calcium, and ferritin measurements. The Fisher-Freeman-Halton exact test was used to determine associations between PPI type or duration and categorical nutrient deficiencies. This test was chosen because some contingency tables had small expected cell counts, making the chi-square test inappropriate. The Kruskal-Wallis test was used to assess differences in continuous nutrient levels across PPI types, and Bonferroni-adjusted post hoc comparisons were used to test for pairwise differences. The chi-square test was used to assess differences in nutrient deficiency rates by sex and age group. The assumptions of independence, mutually exclusive categories, and sufficient expected cell counts were satisfied. The level of statistical significance was determined as p<0.05. Records with incomplete laboratory data for any of the studied micronutrients were excluded during data extraction. Therefore, no statistical imputation for missing data was performed.

Ethical considerations

The research was performed in accordance with the ethical principles of the Declaration of Helsinki. The study was conducted with the Institutional Review Board (IRB) of Pakistan Institute of Medical Sciences (PIMS/IEC/2025/PP-018) and with its moral approval. The study was conducted using existing medical records, and the IRB granted a waiver of written informed consent due to the study's retrospective nature. The study maintained participants' confidentiality by de-identifying personal information, as all data were stored securely and accessible only to official study personnel.

## Results

Table 1 indicates that the study involved 255 participants, among whom 116 (45.5%) were male patients, and 139 (54.5%) were female patients.

**Table 1:** Baseline Characteristics of the study population (n=255) CKD: chronic kidney disease; PPI: proton pump inhibitor.

Variable	Category	n (%)
Age group (years)	18–29	14 (5.5)
	30–39	65 (25.5)
	40–49	65 (25.5)
	50–59	50 (19.6)
	≥60	61 (23.9)
Sex	Male	116 (45.5)
	Female	139 (54.5)
BMI category	Normal	108 (42.4)
	Overweight	86 (33.7)
	Obese	61 (23.9)
Smoking status	Never	147 (57.6)
	Former	79 (31.0)
	Current	29 (11.4)
PPI type	Omeprazole	101 (39.6)
	Pantoprazole	64 (25.1)
	Esomeprazole	61 (23.9)
	Lansoprazole	29 (11.4)
PPI duration	6–12 months	79 (31.0)
	1–3 years	65 (25.5)
	>3 years	111 (43.5)
Dose	Standard	144 (56.5)
	High	111 (43.5)
Comorbidities	None	123 (48.2)
	Hypertension	53 (20.8)
	Diabetes	35 (13.7)
	CKD	29 (11.4)
	Liver disease	15 (5.9)
Concomitant medications	None	159 (62.4)
	Metformin	35 (13.7)
	Diuretics	61 (23.9)

The age distribution of the participants was as follows: 14 (5.5%) aged 18-29 years, 65 (25.5%) aged 30-39 years, 65 (25.5%) aged 40-49 years, 50 (19.6%) aged 50-59 years, and 61 (23.9%) aged 60 and above. In terms of BMI, 108 (42.4%) people were normal, 86 (33.7%) were overweight, and 61 (23.9%) were obese. The majority of the participants were never smokers (147, 57.6%), 79 (31.0%) were former smokers, and 29 (11.4%) were current smokers. The use of PPI involved omeprazole (n=101; 39.6%), pantoprazole (n=64; 25.1%), esomeprazole (n=61; 23.9%), and lansoprazole (n=29; 11.4%). PPI duration was six to 12 months (n=79; 31.0%), one to three years (n=65; 25.5%), and over three years (n=111; 43.5%). PPI doses were either standard (n=144; 56.5%) or high (n=111; 43.5%). Hypertension, diabetes, chronic kidney disease, and liver disease were found among 53 (20.8%), 35 (13.7%), 29 (11.4%), and 15 (5.9%) participants, respectively, and almost half (n=123, 48.2%) of them had no comorbidities. In 159 (62.4%) participants, there were no concomitant medications; with metformin in 35 (13.7%) and diuretics in 61 (23.9%) participants.

The Shapiro-Wilk test indicated that all continuous variables were non-normally distributed (Table 2).

**Table 2:** Normality testing for continuous variables (n=255) The Shapiro–Wilk test is used to assess normality. p<0.05 indicates non-normal distribution.

Variable	Shapiro–Wilk p-value	Distribution
Serum vitamin B12	<0.001	Non-normal
Serum magnesium	0.002	Non-normal
Serum calcium	0.018	Non-normal
Serum ferritin	<0.001	Non-normal

In particular, serum vitamin B12 (p=0.001), serum ferritin (p=0.001), serum magnesium (p=0.002), and serum calcium (p=0.018) had p-values less than 0.05 and were thus significantly different.

Table 3 shows that the deficiency rates for vitamin B12, magnesium, and calcium differed significantly across PPI types.

**Table 3:** Association between the PPI type and nutrient deficiencies (n=255) Fisher–Freeman–Halton exact test used for group comparisons; PPI: Proton pump inhibitors.

Nutrient deficiency	Omeprazole (n=101)	Pantoprazole (n=64)	Esomeprazole (n=61)	Lansoprazole (n=29)	Exact p-value
Vitamin B12	39 (38.6%)	31 (48.4%)	36 (59.0%)	15 (51.7%)	0.018
Magnesium	12 (11.9%)	19 (29.7%)	24 (39.3%)	10 (34.5%)	0.004
Calcium	8 (7.9%)	14 (21.9%)	13 (21.3%)	9 (31.0%)	0.021
Iron/Ferritin	33 (32.7%)	26 (40.6%)	29 (47.5%)	11 (37.9%)	0.089

The highest prevalence of vitamin B12 deficiency was found among participants who used esomeprazole (n=36, 59.0%), and the lowest prevalence was among those who used omeprazole (n=39, 38.6%), with a significant correlation (p=0.018). Likewise, magnesium deficiency was most prevalent in the esomeprazole group (n=24, 39.3%) and least prevalent in the omeprazole group (n=12, 11.9%), with a statistically significant association between PPI types (p=0.004). The PPI type was also significantly associated with calcium deficiency (p=0.021); the highest percentage was in lansoprazole users (n=9, 31.0%), and the lowest was in omeprazole users (n=8, 7.9%). On the other hand, the deficiency in iron/ferritin was not significantly different across PPI types, although prevalence rates varied across groups (p=0.089).

Table 4 demonstrates that the prevalence of iron/ferritin deficiency was almost identical in all PPI duration categories.

**Table 4:** Association between duration of PPI use and iron/ferritin deficiency (n=255) Fisher–Freeman–Halton exact test, p=0.12 (not statistically significant); PPI: Proton pump inhibitor.

PPI duration	Iron/Ferritin deficiency (n)	Total	Percentage
6-12 months	39	79	49.4%
1-3 years	32	65	49.2%
>3 years	54	111	48.6%

The participants who were found to be iron/ferritin deficient were 39 out of 79 (49.4%), 32 out of 65 (49.2%), and 54 out of 111 (48.6%), in those using PPIs for six to 12 months, one to three years, and over three years, respectively. According to the Fisher-Freeman-Halton exact test, the association between PPI duration and iron/ferritin deficiency was not statistically significant (p≈0.12).

Table 5 shows that serum calcium levels varied significantly across PPI types.

**Table 5:** Comparison of serum calcium levels across PPI types (n=255) Values are presented as medians and interquartile ranges (IQRs). η²: eta squared effect size; PPI: proton pump inhibitor.

PPI type	Median calcium (mg/dL)	IQR
Omeprazole	9.06	1.05
Pantoprazole	8.94	1.02
Esomeprazole	8.98	0.98
Lansoprazole	8.71	1.11

Omeprazole users had the highest calcium level of 9.06 mg/dL (interquartile range or IQR=1.05), followed by the lowest level of 8.71 mg/dL in users of lansoprazole (IQR=1.11), while the intermediate values in the users of pantoprazole (8.94 mg/dL, IQR=1.02) and esomeprazole users (8.98 mg/dL, IQR = 0.98). The Kruskal-Wallis test showed that the difference in serum calcium across groups was statistically significant (H=11.29, df=3, p=0.010). The effect size was small (η2=0.04), indicating that PPI type explained only a small proportion of the variation in serum calcium levels.

As presented in Table 6, female participants were found to have a very high prevalence of vitamin B12 deficiency (61/139, 43.9%), contrasting with male participants (24/116, 20.7%) (p<0.001).

**Table 6:** Sex differences in nutrient deficiency status (n=255) Calculated via chi-square test.

Nutrient	Male participants	Female participants	p-value
Vitamin B12	24/116 (20.7%)	61/139 (43.9%)	<0.001
Iron/Ferritin	19/116 (16.4%)	112/139 (80.6%)	<0.001
Magnesium	52/116 (44.8%)	59/139 (42.4%)	0.610
Calcium	29/116 (25.0%)	32/139 (23.0%)	0.653

In the same way, the prevalence of iron/ferritin deficiency among female subjects was significantly higher, as compared to male subjects (112/139, 80.6% vs 19/116, 16.4%; p<0.001). These sex differences may act as potential confounders in the observed associations between PPI use and nutrient deficiencies. However, there was no statistically significant sex difference in magnesium deficiency (male subjects: 52/116, 44.8% vs female subjects: 59/139, 42.4%) or calcium deficiency (male subjects: 29/116, 25.0% vs female subjects: 32/139, 23.0%).

Table 7 shows that nutrient-deficit rates differed significantly by age for vitamin B12 (p=0.042), magnesium (p=0.038), and calcium (p=0.027).

**Table 7:** Nutrient deficiency rates by age group (n=255) Data are presented as percentages within each age group. P-values were calculated using the Pearson Chi-square test to compare deficiency rates across age categories. *P<0.05 was considered statistically significant.

Deficiency	18–29 yrs (n=14)	30–39 yrs (n=65)	40–49 yrs (n=65)	50–59 yrs (n=50)	≥60 yrs (n=61)	p-value
Vitamin B12	8/14 (57.1%)	27/65 (41.5%)	29/65 (44.6%)	19/50 (38.0%)	22/61 (36.1%)	0.042*
Magnesium	10/14 (71.4%)	21/65 (32.3%)	28/65 (43.1%)	19/50 (38.0%)	21/61 (34.4%)	0.038*
Calcium	7/14 (50.0%)	16/65 (24.6%)	18/65 (27.7%)	12/50 (24.0%)	13/61 (21.3%)	0.027*
Iron/Ferritin	8/14 (57.1%)	38/65 (58.5%)	36/65 (55.4%)	28/50 (56.0%)	28/61 (45.9%)	0.201

The most common deficiency proportions for the following nutrients were observed in the 18-29 age group: vitamin B12 deficiency (8/14; 57.1%), magnesium deficiency (10/14; 71.4%), and calcium deficiency (7/14; 50.0%). However, the small sample size in this youngest age group limits the reliability and generalizability of these estimates. Iron/ferritin deficiency did not differ significantly across age groups (p=0.201), with similar rates across most groups.

## Discussion

This is a cross-sectional, retrospective study evaluating the relationship between nutrient deficiency and long-term use of PPIs in adult patients. In our research, the prevalence of vitamin B12 deficiency was higher among esomeprazole users (59.0%) than among omeprazole users (38.6%), suggesting a potential association between PPI type and vitamin B12 deficiency. These results are consistent with the current literature and indicate that PPIs can reduce serum vitamin B12 concentrations, impair its absorption, and warrant surveillance of B12 levels in long-term PPI users [11].

In our study, magnesium deficiency was most common among esomeprazole (39.3%) and lansoprazole (34.5%) users compared with omeprazole users (11.9%), suggesting that the type of PPI used may affect the risk of magnesium deficiency. This is consistent with a meta-analysis of multiple studies that showed that PPI use leads to an overall increased risk of hypomagnesemia (median prevalence 27.1%, pooled OR 1.775) [12]. In our research, the prevalence of calcium deficiency differed between omeprazole and lansoprazole users (p=0.021). While statistically significant, this finding also has potential clinical relevance, as a higher rate of calcium deficiency may increase the risk of bone loss or secondary hyperparathyroidism among lansoprazole users. This is consistent with prior literature, which has established that PPI use correlates with hyperparathyroidism, likely due to calcium malabsorption (OR 1.56, p=0.018) [13]. Iron/ferritin deficiency varied by 32.7% and 47.5% in omeprazole and esomeprazole groups, respectively (p=0.089), suggesting that the type of PPI did not significantly affect iron status. This is in line with past studies suggesting that both omeprazole and esomeprazole, and not pantoprazole, are associated with a greater risk of iron deficiency, which confirms the trend in our cohort [14].

Iron/ferritin deficiency did not differ significantly with PPI duration in our cohort (p≈0.12). In line with this, earlier research has shown that PPI use is associated with an increased risk of iron deficiency, especially at higher doses, and that impaired intestinal iron absorption is just one possible mechanism [15]. Similarly, our finding of increased median calcium levels among omeprazole users is corroborated by experimental research demonstrating type-specific effects of PPIs on calcium metabolism [16].

Regarding sex-specific differences, vitamin B12 and iron/ferritin deficiencies were observed more frequently in female subjects, consistent with the existing literature on physiological needs and dietary factors. However, these findings are unadjusted for potential confounders such as age, PPI type, dose, and comorbidities, and should therefore be interpreted as associations rather than causal effects [17,18]. Magnesium and calcium deficits did not differ significantly between male participants and female participants in our cohort. This is consistent with previous research, which found that levels of magnesium and calcium uptake and deficiency did not differ significantly by sex [19].

In line with earlier research, our results showed a significant association between age groups and vitamin B12 deficiency (p=0.042). Surprisingly, though the lack of folate was more pronounced in the older population studied, the most significant deficit was found in our cohort in younger populations, suggesting that specific populations may be more susceptible to poor diet or lifestyle factors [20,21]. In our study, the prevalence of magnesium deficiency was highest in the youngest age group and decreased with age. This is in contrast to previous reports that suggest magnesium deficiency is more common among older people, suggesting a possible influence of dietary habits or lifestyle factors in our society [22]. In our study, results indicated greater calcium deficiency in younger adults. In contrast, in previous population-based studies with lower calcium intake, the opposite was observed in older adults. Such a discrepancy can be explained by regional dietary habits, lifestyle factors, or supplementation practices in our cohort [23]. In our study, the prevalence of iron/ferritin deficiency was relatively high across all age groups, with no significant difference between age groups. This result is in line with a previous survey, which found that functional iron deficiency occurs among adults of all ages and, therefore, age does not appear to be a significant factor in determining iron deficiency in the general adult population [24].

All in all, these findings emphasize the effects of PPI type, sex, and age on nutrient deficiencies, as well as population-specific patterns in magnesium, calcium, and B12 deficiencies. Nutrient monitoring and personalized diet or supplementation plans may be warranted for long-term PPI users, especially among younger adults and women.

Limitations

Several limitations must be considered when interpreting the results. First, the retrospective observational design limits causal inference; observed associations cannot confirm a direct effect of PPI use on nutrient deficiencies. Second, the study relied on electronic medical records and laboratory databases, which may contain incomplete information and introduce selection bias, particularly among patients with missing laboratory data. Third, the timing of laboratory measurements relative to PPI exposure may vary, introducing temporal ambiguity. Fourth, the relatively small sample sizes in certain subgroups may limit statistical power to detect differences. Fifth, institutional laboratory reference ranges were used to define deficiencies, and clinical manifestations (e.g., anemia severity, neurological symptoms, or bone health outcomes) were not assessed. Sixth, dietary intake, socioeconomic status, and supplement use were not fully captured, which may have contributed to residual confounding. Finally, there was no non-PPI control group, preventing a direct comparison with the general population or with patients receiving alternative therapies, such as H2 receptor antagonists. Also, data from both centers were pooled, and center-specific effects were not analyzed.

Future directions

Future research should focus on prospective longitudinal studies to better establish temporal relationships between long-term PPI exposure and nutrient depletion. Studies that incorporate control groups of people who do not use PPIs and people who use H2 receptor antagonists will help researchers determine whether deficiencies result from PPIs or from existing gastrointestinal diseases and preexisting nutritional vulnerabilities. Future studies with larger sample sizes are recommended to validate and expand upon these findings. Increasing the number of participants, especially in subgroups defined by PPI type, duration, or age, would strengthen the statistical power and enhance the clinical applicability of the results.

Further studies are needed to examine how different doses affect outcomes and to evaluate the effectiveness of deprescribing methods to restore lost nutrients. Randomized studies that test multiple PPI agents will show which PPIs pose greater risks to nutritional health. Future research should link clinical outcomes of anemia severity, bone mineral density, fractures, and neurocognitive symptoms to determine which laboratory-confirmed deficiencies have real clinical significance. Research on targeted monitoring protocols and preventive supplementation methods for high-risk patients will provide practical solutions for everyday clinical practice.

## Conclusions

In this retrospective observational study of adult patients receiving long-term PPI therapy, a high prevalence of vitamin B12, magnesium, and calcium deficiencies was observed. Nutrient deficiencies varied significantly according to PPI type, with esomeprazole and lansoprazole users demonstrating comparatively higher deficiency rates for certain micronutrients. In contrast, iron/ferritin deficiency remained consistently prevalent across all PPI types and durations, suggesting a more complex or multifactorial relationship. Additionally, significant differences by age and sex highlight the presence of potentially vulnerable subgroups, particularly younger adults and female patients.

These findings underscore the importance of routine monitoring of micronutrient status in patients on prolonged PPI therapy. Clinicians should consider individualized risk assessment, appropriate laboratory surveillance, and targeted supplementation where necessary. Future prospective studies are warranted to clarify dose-response relationships, establish causality, and evaluate the effectiveness of deprescribing strategies in mitigating long-term nutritional complications.
